# Prognostic Significance of +1q Alterations in Relapsed/Refractory Multiple Myeloma Treated With Daratumumab‐, Elotuzumab‐, and Carfilzomib‐Based Triplet Regimens: A Multicenter Real‐World Analysis of 635 Patients

**DOI:** 10.1111/ejh.14413

**Published:** 2025-03-19

**Authors:** Fortunato Morabito, Enrica Antonia Martino, Monica Galli, Massimo Offidani, Renato Zambello, Sara Bringhen, Nicola Giuliani, Catello Califano, Marino Brunori, Alfredo Gagliardi, Nicola Sgherza, Angela Maria Quinto, Gregorio Barilà, Angelo Belotti, Claudio Cerchione, Gloria Margiotta Casaluci, Raffaele Fontana, Velia Bongarzoni, Giuseppe Tarantini, Daniele Derudas, Francesca Patriarca, Alessandro Gozzetti, Adelina Sementa, Elisabetta Antonioli, Angela Rago, Flavia Lotti, Claudio De Magistris, Maria Teresa Petrucci, Loredana Pettine, Niccolò Bolli, Concetta Conticello, Elena Zamagni, Salvatore Palmieri, Maurizio Musso, Anna Mele, Roberta Della Pepa, Ernesto Vigna, Antonella Bruzzese, Francesca Fazio, Roberto Mina, Laura Paris, Iolanda Donatella Vincelli, Giuliana Farina, Clotilde Cangialosi, Katia Mancuso, Antonietta Pia Falcone, Giuseppe Mele, Antonello Sica, Sonia Morè, Giovanni Reddiconto, Giovanni Tripepi, Graziella D'Arrigo, Emiliano Barbieri, Micol Quaresima, Claudio Salvatore Cartia, Sara Pezzatti, Magda Marcatti, Francesca Farina, Anna Cafro, Michele Palumbo, Valeria Masoni, Virginia Valeria Ferretti, Francesco Di Raimondo, Pellegrino Musto, Antonino Neri, Silvia Mangiacavalli, Massimo Gentile

**Affiliations:** ^1^ Gruppo Amici Dell'Ematologia Foundation‐GrADE Reggio Emilia Italy; ^2^ Hematology Unit AO of Cosenza Cosenza Italy; ^3^ Hematology and Bone Marrow Transplant Unit ASST Papa Giovanni XXIII Bergamo Italy; ^4^ Hematology Unit Ancona Italy; ^5^ Department of Medicine (DIMED), Hematology and Clinical Immunology Padua University School of Medicine Padova Italy; ^6^ Division of Hematology, AOU Città Della Salute e Della Scienza di Torino University of Torino Torino Italy; ^7^ Hematology Unit Parma University Hospital Parma Italy; ^8^ Onco‐Hematology Unit “A. Tortora” Hospital Pagani Italy; ^9^ Internal Medicine Ospedale Santa Croce Fano Italy; ^10^ Hematology, “Santa Maria di Loreto Nuovo” Hospital Naples Italy; ^11^ Unit of Hematology and Stem Cell Transplantation Bari Italy; ^12^ Haematology and Transplant Unit IRCCS—Istituto Tumori “Giovanni Paolo II” Bari Italy; ^13^ Hematology Unit Ospedale San Bortolo Vicenza Italy; ^14^ Hematology Unit A.O. Spedali Civili Brescia Italy; ^15^ Department of Hematology IRCCS Istituto Scientifico Romagnolo per Lo Studio e la Cura Dei Tumori (IRST) Cesena Italy; ^16^ Division of Hematology, Department of Translational Medicine University of Eastern Piedmont Novara Italy; ^17^ Hematology and Transplant Center University Hospital “San Giovanni di Dio e Ruggi d'Aragona” Salerno Italy; ^18^ UOC of Hematology San Giovanni‐Addolorata Hospital Rome Rome Italy; ^19^ Hematology Unit, “Dimiccoli” Hospital Barletta Italy; ^20^ Department of Hematology Cagliari Italy; ^21^ Hematology, DMED University of Udine, University Hospital of Friuli Centrale Udine Italy; ^22^ Department of Medicine, Surgery and Neurosciences University of Siena Policlinico S Maria Alle Scotte Siena Italy; ^23^ UOC Ematologia Ospedale San Giuseppe Moscati Avellino Italy; ^24^ Haematology Unit Careggi University Hospital Florence Italy; ^25^ UOSD Ematologia ASL Roma 1 Rome Italy; ^26^ Institute of Hematology TMO Azienda Universitaria‐Ospedaliera Santa Maria Della Misericordia di Perugia Perugia Italy; ^27^ Hematology Unit Fondazione IRCCS Ca' Granda Ospedale Maggiore Policlinico Milan Italy; ^28^ Department of Translational and Precision Medicine Hematology Azienda Policlinico Umberto I Sapienza University of Rome Rome Italy; ^29^ Division of Hematology, Azienda Policlinico‐OVE University of Catania Catania Italy; ^30^ IRCCS Azienda Ospedaliero‐Universitaria di Bologna Istituto di Ematologia “Seràgnoli” Bologna Italy; ^31^ Dipartimento di Medicina Specialistica, Diagnostica e Sperimentale Università di Bologna Bologna Italy; ^32^ Hematology Unit Napoli Italy; ^33^ UOC OncoEmatologia e TMO Dipartimento Oncologico Palermo Italy; ^34^ Department of Hematology and Bone Marrow Transplant Hospital Card. G. Panico Lecce Italy; ^35^ Hematology AUOP “Federico II” Naples Italy; ^36^ Hematology Unit, Department of Hemato‐Oncology and Radiotherapy Great Metropolitan Hospital “Bianchi‐Melacrino‐Morelli” Reggio Calabria Italy; ^37^ UOC Ematologia a Indirizzo Oncologico AORN “Sant'Anna e San Sebastiano” Caserta Italy; ^38^ UOC Ematologia A. O. Ospedali Riuniti Villa Sofia‐Cervello Palermo Italy; ^39^ Department of Hematology and Bone Marrow Transplant IRCCS Casa Sollievo Della Sofferenza San Giovanni Rotondo Italy; ^40^ Department of Hematology Hospital Perrino Brindisi Italy; ^41^ Onco‐Hematology AOU “Vanvitelli” Naples Italy; ^42^ Department of Hematology Hospital Vito Fazzi Lecce Italy; ^43^ CNR‐IBIM Clinical Epidemiology and Physiopathology of Renal Diseases and Hypertension of Reggio Calabria Reggio Calabria Italy; ^44^ Hematology Unit Azienda USL‐IRCCS di Reggio Emilia Emilia Romagna Italy; ^45^ Clinical and Experimental Medicine PhD Program University of Modena and Reggio Emilia Modena Italy; ^46^ Division of Hematology IRCCS Fondazione Policlinico San Matteo Pavia Italy; ^47^ Division of Hematology San Gerardo Hospital Monza Italy; ^48^ Division of Hematology and Bone Marrow Transplant Unit IRCCS San Raffaele Scientific Institute Milan Italy; ^49^ Department of Molecular Medicine University of Pavia Pavia Italy; ^50^ Clinical Epidemiology and Biostatistics Service Fondazione IRCCS Policlinico San Matteo Pavia Italy; ^51^ Scientific Directorate IRCCS of Reggio Emilia Reggio Emilia Italy; ^52^ Department of Pharmacy, Health and Nutritional Science University of Calabria Rende Italy

**Keywords:** +1q alterations and prognosis in RRMM 1q abnormalities, DaraRd, EloPd, EloRd, KRd, RRMM

## Abstract

Relapsed/refractory multiple myeloma (RRMM) research on the impact of +1q abnormalities in real‐world settings is limited. This study evaluated the prognostic and predictive significance of 1q gain [gain(1q)] and amplification [ampl(1q)] in 635 RRMM patients treated with daratumumab‐, elotuzumab‐, and carfilzomib‐based triplet regimens. Patients with +1q abnormalities had lower deep response rates [≥ CR: 9.4% for gain(1q), 11.6% for ampl(1q)] versus 20.2% in +1q‐negative patients. Multivariable ordinal logistic analysis showed significantly lower odds of achieving ≥ CR in patients with gain(1q) (OR = 0.49, *p* < 0.001) or ampl(1q) (OR = 0.58, *p* = 0.0037). Progression‐free survival (PFS) was longer in +1q‐negative patients (28 months) compared to those with gain(1q) (8 months) or ampl(1q) (7.4 months). Multivariable models identified gain(1q) (HR = 1.9, *p* < 0.001) and ampl(1q) (HR = 2.2, *p* < 0.001) as independent negative prognostic factors alongside del17p, *t*(4;14), creatinine clearance < 60 mL/min, and ISS Stages II and III. Similarly, overall survival (OS) was reduced for patients with gain(1q) (25 months) and ampl(1q) (19.5 months) versus 42.2 months in +1q‐negative patients. Multivariable analysis showed gain(1q) (HR = 1.6, *p* = 0.007) and ampl(1q) (HR = 2.0, *p* = 0.002) as independent predictors of increased mortality. Ancillary +1q abnormalities associated with high‐risk cytogenetic changes were linked to both shorter PFS and OS. Stratification into no‐hit, single‐hit, double‐hit, and triple‐hit groups showed significant survival differences, emphasizing the impact of cumulative cytogenetic abnormalities on outcomes. In conclusion, +1q abnormalities significantly impact prognosis in RRMM and should be considered in risk stratification. The study emphasizes the importance of comprehensive cytogenetic profiling in real‐world settings and highlights the need for personalized treatment strategies to improve patient outcomes.

## 1 Introduction

Multiple myeloma (MM) is a plasma cell malignancy characterized by significant clinical and biological complexity. It accounts for approximately 1% of all cancers and roughly 10% of hematologic malignancies, predominantly affecting older individuals. The disease consistently arises from precursor states, which represent early, often asymptomatic stages of its evolution [1].

Relapsed and refractory multiple myeloma (RRMM) remains a significant therapeutic challenge in the management of the disease [1]. Triplet regimens incorporating newer agents such as proteasome inhibitor carfilzomib, the second‐generation immunomodulating agents (IMiDs) like pomalidomide, and anti‐CD38 monoclonal antibodies such as daratumumab have shown substantial efficacy [2], even in the real‐world setting [3, 4, 5, 6, 7, 8]. More recently, innovative therapies like chimeric antigen receptor T (CAR‐T) cell therapy and bispecific antibodies targeting BCMA have emerged as promising options [2, 9, 10, 11], particularly for patients who have exhausted conventional treatments [12].

At the molecular level, MM is driven by a spectrum of genetic abnormalities that influence its pathogenesis, clinical presentation, and prognosis [1, 13, 14, 15]. Primary cytogenetic abnormalities, such as translocations involving the immunoglobulin heavy chain (IgH) locus on chromosome 14 and trisomies of odd‐numbered chromosomes, act as initiating events. These abnormalities frequently define distinct molecular subtypes of MM, such as *t*(11;14), *t*(4;14), or hyperdiploid MM, each with unique clinical and biological characteristics. As the disease progresses, secondary cytogenetic changes, including +1q alterations [16, 17, 18, 19], commonly emerge and are often associated with worse outcomes. These molecular insights have led to the redefinition of MM as a group of related but distinct diseases rather than a single entity.

Effective risk stratification is pivotal for prognostic assessment and tailoring therapeutic strategies in MM. The International Staging System (ISS) [20] and its revised version [21] integrate critical cytogenetic markers of high‐risk disease, such as *t*(4;14), *t*(14;16), and del(17p). Chromosome 1q abnormalities, including gain(1q) and amplification [amp(1q)], are particularly prevalent and clinically significant [16, 17, 18, 19], affecting approximately 40% of newly diagnosed MM cases and up to roughly 70% of RRMM cases [22]. However, the reported incidence may vary depending on the cutoff thresholds used to define positivity for 1q abnormalities, highlighting the importance of standardized criteria in their detection and reporting.

Chromosome 1q abnormalities are pivotal in the pathogenesis and progression of MM, representing a significant area of research [reviewed in 16–19]. Overexpression of genes on the 1q region has been strongly associated with unfavorable outcomes in MM, highlighting its potential role in disease progression and therapeutic resistance [23]. Genes such as CKS1B, PSMD4, ADAR1, and MCL1 have been implicated in MM progression, contributing to cell cycle disruption, proteasome regulation, and resistance to apoptosis. Despite these associations, it remains uncertain whether +1q alterations act as primary drivers of poor prognosis or if they reflect broader genomic instability [24]. Biologically, although both the gain(1q) and ampl(1q) lead to overexpression of critical oncogenes, the resultant downstream effects seem to be more pronounced in ampl(1q), which is associated with clonal evolution, higher relapse rates, and inferior survival outcomes. Nevertheless, clinically, +1q abnormalities have been incorporated into updated risk stratification systems, such as the second revision of the ISS (R2‐ISS) [25].

The prognostic relevance of +1q abnormalities has been extensively studied in patients with newly diagnosed MM, within the context of first‐line therapy, underscoring its role in influencing disease outcomes ([26, 27, 28, 29, 30, 31, 32] reviewed in [33]). However, its clinical impact in RRMM remains less well understood [33, 34], where therapeutic options and disease biology differ significantly.

This study addresses this gap by evaluating the prognostic implications of +1q abnormalities in RRMM patients undergoing various salvage regimens. Utilizing real‐world data, it aims to clarify the predictive value of +1q in determining treatment responses, progression‐free survival (PFS), and overall survival (OS). Ultimately, this study aims to enhance risk stratification and guide personalized therapeutic approaches for this high‐risk population, contributing to improved outcomes in MM management.

## 2 Methods

### 2.1 Patients

This retrospective, multicenter analysis included patients with RRMM treated at 51 centers across Italy. Clinical data were extracted from medical records and consolidated into a centralized database, capturing variables such as age, gender, diagnosis date, laboratory parameters, treatment history, and date of last follow‐up or death. Data collection commenced at the time of inclusion and was updated with subsequent follow‐up information. Of the initial cohort of 1556 RRMM patients, 921 were excluded due to missing information regarding +1q abnormality. Thus, 635 patients fulfilled the study's inclusion criteria (Figure S1), with documented +1q chromosomal alteration status at the initiation of salvage therapy.

The 1q21+ abnormality is defined as a gain of 1q21 (gain[1q21], 3 copies) and amplification of 1q21 (amp[1q21], ≥ 4 copies) in ≥ 30% of CD138+ plasma cells analyzed for cytogenetic risk evaluation [35].

Patients received salvage therapy, consisting of at least one cycle of elotuzumab with lenalidomide and dexamethasone (Elo‐Rd, 67 patients), or elotuzumab with pomalidomide and dexamethasone (Elo‐Pd, 131 patients), or carfilzomib with Rd (KRd, 296 patients), or daratumumab with Rd (DaraRd, 141 patients) between January 2021 and June 2024 in routine clinical practice. Treatments were administered according to approved indications and prescribing guidelines.

During therapy, prophylactic antibacterial, antiviral, and antithrombotic measures were routinely implemented following each center's policies. Treatment continued until disease progression, the onset of unacceptable toxicity, or withdrawal of patient consent.

Time‐to‐event endpoints included PFS and OS. Treatment responses and disease progression were assessed based on International Myeloma Working Group (IMWG) criteria [36, 37]. A treatment response was defined as achieving at least partial remission (PR). Refractory myeloma was defined as a disease unresponsive to primary or salvage therapy or progression within 60 days of the last treatment.

The study protocol was reviewed and approved by Institutional Ethics Committees, adhering to the principles of the Declaration of Helsinki. All patients enrolled have signed the informed consent.

### 2.2 Statistical Analysis

Categorical variables were analyzed using Fisher's exact test for two‐way tables and Pearson's chi‐square test for contingency tables with more than two categories.

Multivariable ordinal regression was employed to evaluate potential confounders influencing the association between treatment response and variables that were significant in univariable analysis (Pearson's chi‐square or Fisher's exact test). In the ordinal logistic regression analysis, the proportionality of odds was formally tested by the likelihood ratio test (LR test) and data were expressed as odds ratios (ORs), 95% confidence interval (CI), and *p* values.

Kaplan–Meier method was used to evaluate PFS and OS. PFS was measured from the initiation of RRMM treatment to disease progression, initiation of subsequent therapy, or last follow‐up, and OS was measured until death from any cause or last follow‐up. The optimal age threshold to predict death was determined using a receiver operating characteristic (ROC) curve analysis. The Youden index was used to determine the optimal age cut‐off that maximized the difference between the true positive rate (sensitivity) and the false positive rate (1 − specificity).

Associations between individual variables and survival outcomes were tested using the log‐rank test. Prognostic variables were further examined through univariable and multivariable Cox regression analyses, with results expressed as hazard ratios (HR) and 95% CIs. Statistical significance was set at *p* ≤ 0.05.

All statistical analyses were performed using STATA for Windows (v.9) and SPSS Statistics (v.21).

## 3 Results

### 3.1 Patient Characteristics

The baseline characteristics of the 635 RRMM patients are depicted in Table 1.

**TABLE 1:** Baseline characteristics of 635 relapsed/refractory multiple myeloma (RRMM) patients treated with KRd, DaraRd, EloRd, or EloPd in a real‐world setting at therapy initiation.

	No. of patients (%)				
	All cases (*N* = 635)	+1q negative (*N* = 469)	1q gain (*N* = 106)	1q amplification (*N* = 60)	*p*
Age, (years) median (range)	68 (34–89)				
Gender					
Male	334 (52.6)	241 (72.2)	57 (17.1)	36 (10.8)	0.43
Female	301 (47.4)	228 (75.7)	49 (16.3)	24 (8)	
Paraproteins (isotype)					
Immunoglobulin G	375 (59.1)	280 (74.7)	60 (16)	35 (9.3)	0.13
Immunoglobulin A	136 (21.4)	90 (66.2)	30 (22.1)	16 (11.8)	
Immunoglobulin D	9 (1.4)	5 (55.6)	4 (44.4)	0 (0)	
Immunoglobulin M	4 (0.6)	4 (100)	0 (0)	0 (0)	
Light chain only	104 (16.4)	84 (80.8)	11 (10.6)	9 (8.7)	
Non secretory	7 (1.1)	6 (85.7)	1 (14.3)	0 (0)	
Creatinine clearance (mL/min)					
≥ 60	394 (62)	305 (77.4)	57 (14.5)	32 (8.1)	0.034
< 60	241 (38)	164 (68)	49 (20.3)	28 (11.6)	
International staging system					
I	250 (39.4)	201 (80.4)	35 (14)	14 (5.6)	0.006
II	249 (39.2)	181 (72.7)	40 (16.1)	28 (11.2)	
III	136 (21.4)	87 (64)	31 (22.8)	18 (13.2)	
*t*(4;14)					
Negative	561 (88.3)	423 (75.4)	86 (15.3)	52 (9.3)	0.029
Positive	74 (11.7)	46 (62.2)	20 (27)	8 (10.8)	
*t*(14;16)					
Negative	620 (95.7)	462 (74.5)	101 (16.3)	57 (9.2)	0.05
Positive	15 (4.3)	7 (46.7)	5 (33.3)	3 (20)	
del1p					
Negative	607 (95.6)	453 (74.6)	97 (16)	57 (9.4)	0.061
Positive	27 (4.2)	16 (59.3)	9 (33.3)	2 (7.4)	
Missing	1 (0.2)	0 (0)	0 (0)	1 (100)	
del17p					
Negative	554 (87.2)	409 (73.8)	94 (17)	51 (9.2)	0.79
Positive	80 (12.6)	59 (73.8)	12 (15)	9 (11.3)	
Missing	1 (0.2)	1 (100)	0 (0)	0 (0)	
Previous lines of therapy					
1	377 (59.4)	298 (79)	52 (13.8)	27 (7 0.2)	0.006
2	148 (23.3)	102 (68.9)	28 (18.9)	18 (12.2)	
≥ 2	110 (17.3)	69 (62.7)	26 (23.6)	15 (13.6)	
Previous autologous stem cell transplantation					
No	366 (57.6)	267 (73)	63 (17.2)	36 (9.8)	0.83
Yes	269 (42.4)	202 (75.1)	43 (16)	24 (8.9)	
Disease status					
Relapse	310 (48.8)	216 (69.7)	60 (19.4)	34 (11)	0.36
Refractory	234 (36.9)	175 (74.8)	35 (15)	24 (10.3)	
Missing	91 (14.3)	78 (85.7)	11 (12.1)	2 (2.2)	
Last therapy					
KRd	296 (46.6)	216 (73)	44 (14.9)	33 (12.2)	0.001
DaraRd	141 (22.2)	118 (83.7)	20 (14.2)	3 (2.1)	
EloPd	131 (20.6)	83 (63.4)	30 (22.9)	18 (13.7)	
EloRd	67 (10.6)	52 (77.6)	12 (17.9)	3 (4.5)	

The median age at therapy initiation was 68 years (range 34–89), and the cohort exhibited a balanced gender distribution, with 52.6% male and 47.4% female patients. Paraprotein subtype analysis showed a predominance of IgG (59.1%), followed by IgA (21.4%), while rarer subtypes such as IgD (1.4%) and IgM (0.6%) were infrequent; 16.4% of patients had light chain‐only disease. Renal function, measured as creatinine clearance, revealed that 38% of patients had impaired renal function (< 60 mL/min) at baseline. Disease staging using the ISS, evaluated at the salvage therapy start, indicated that nearly 80% of patients were in Stages I or II, while 21.4% had advanced‐stage disease (Stage III). Chromosomal abnormalities were present in most cases, with +1q abnormalities representing the most common abnormality observed in 26.1% of patients [gain(1q): 16.7%, ampl(1q): 9.4%]. Other high‐risk cytogenetic features included *t*(4;14) (11.7%), *t*(4;16) (4.3%), del17p (12.6%), and del1p (2.4%) with no cases of biallelic del1p detected. The cohort had predominantly received limited prior lines of therapy, with 59.4% of patients treated with one previous line and 23.3% with two. There were 17.3% of patients who had undergone three or more lines of therapy. Notably, 42.4% had received a prior autologous stem cell transplant. In terms of treatment allocation, the most frequently administered regimen was KRd (46.6%), followed by DaraRd (22.2%), EloPd (20.6%), and EloRd (10.6%). Regarding disease status, 57% of patients were relapsed, while 43% were refractory.

The distribution of +1q abnormalities revealed a significantly higher prevalence of gain(1q) in patients with impaired renal function (CrCl < 60 mL/min), ISS Stage III, and co‐occurring high‐risk cytogenetic abnormalities such as *t*(4;14) and *t*(14;16), as well as in those who have received > 2 previous lines of therapy (Table 1). Additionally, +1q negative status was more frequently observed in patients who received DaraRd as the last therapy, while ampl(1q) was more common in patients who received EloPd (Table 1).

The limited availability of FISH analysis for 1q abnormalities in only 42.1% of the cohort (635/1556 cases), although sufficiently large to enable a meaningful evaluation of the clinical impact of 1q abnormalities, could represent a limitation of this study. To minimize the potential for selection bias and strengthen the robustness and generalizability of our findings, the baseline characteristics of patients with and without available +1q data were compared (Table S4). This analysis showed no significant differences between the two groups, underscoring the representativeness of the subgroup with available +1q abnormality analysis for the overall cohort.

### 3.2 Response Evaluation

The ORR was 78.1%, with 13 (2%) stringent complete responses (sCRs), 99 (15.6%) CRs, 197 (31%) very good partial responses (VGPRs), and 187 (29.4%) partial responses (PRs), while the remaining 139 (21.9%) cases were classified as minimal response, stable disease, and progressive disease (< PRs).

The distribution of responses, ranging from CR or better to less than PR, varied significantly across the variables indicated in Table S4, revealing a complex relationship between clinical, biological, and treatment‐related factors. Patients without +1q abnormalities achieved ≥ CR in 20.2% of cases, while those with gain(1q) or ampl(1q) had lower rates of deep responses (9.4% and 11.6%, respectively). Moreover, achieving CR or better was significantly associated with fewer prior lines of therapy. Specifically, 22.8% of patients who had received only one prior line of therapy achieved ≥ CR, compared to 12.1% and 7.2% for those with two or more lines, respectively. Similarly, cytogenetic abnormalities played a significant role, with patients negative for del17p achieving ≥ CR in 18% of cases compared to 15% among those with del17p positivity. A similar trend was observed for *t*(4;14), where ≥ CR rates were 18.7% in negative cases versus 9.4% in positive ones. Therapeutic regimens also had a marked impact on response rates. Among the treatments analyzed, DaraRd and KRd resulted in the highest proportion of deep responses, with 24.8% and 23.3% achieving ≥ CR and 37.5% and 32% VGPR, respectively, whereas EloPd showed markedly lower rates of ≥ CR (1.5%) and VGPR (17.5%). Disease stage further influenced outcomes, as 20.8% of patients with ISS I achieved ≥ CR, compared to 16% with ISS II and 14.7% with ISS III, illustrating the predictive value of disease burden at diagnosis. The type of monoclonal component was another significant factor, with patients producing IgG or IgA achieving lower rates of ≥ CR (15.2% and 15.4%, respectively) compared to those with light chains only (26.9%) or other components (30%). Despite these significant findings, gender, renal function, specific cytogenetic markers such as del1p and *t*(14;16), and age held no significant impact on therapy response.

The effect of 1q alteration on response was assessed using multivariable ordinal logistic regression analysis having as the dependent variable the response grouped as follows: 3 ≥ CR; 2 = VGPR; 1 = PR; 0 = other response. The results of this analysis (Table S4) indicated that patients with gain(1q) (OR = 0.49, *p* < 0.001) and ampl(1q) (OR = 0.58, *p* = 0.037) had significantly lower odds of achieving ≥ CR compared to those who were +1q negative, independently of ISS, type of monoclonal component, *t*(4:14), last therapy, del17, and lines of therapy. Moreover, ISS (*p* = 0.013), type of monoclonal component (other [no IgG and no IgA] vs. IgG *p* = 0.021), last therapy (EloPD, EloRD vs. DaraRd, *p* < 0.001 and *p* = 0.020, respectively), del17 (*p* = 0.019), and lines of therapy (*p* < 0.001) showed a statistically significant independent negative impact on achieving ≥ CR.

### 3.3 Progression‐Free Survival

**FIGURE 1:**
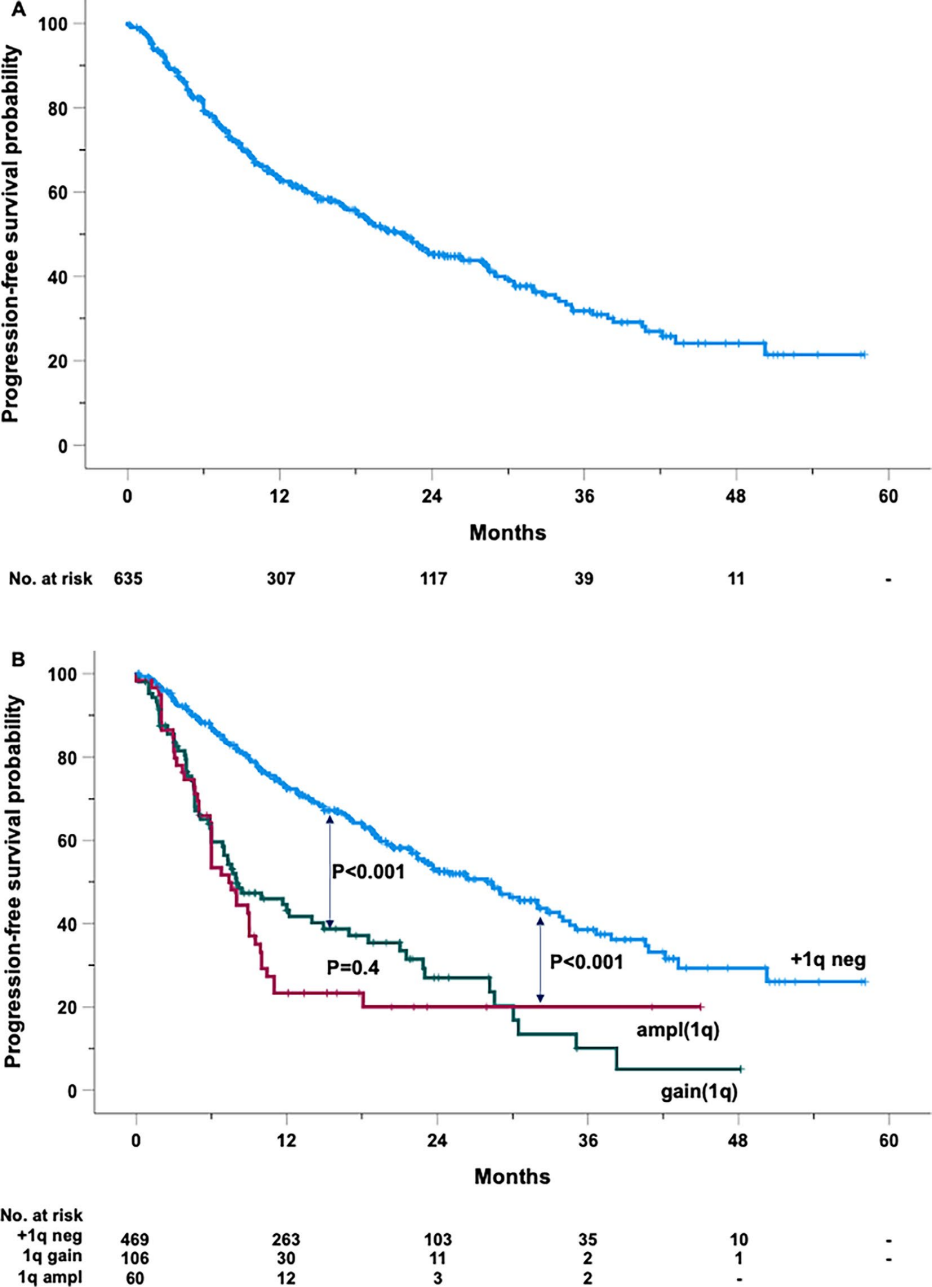
Kaplan–Meier curve of progression‐free survival of the entire cohort (A) and by the absence (+1q neg) or the presence of 1q amplification (ampl1q) or gain (gain1q) (B).

After a median follow‐up of 21 months (range 1–58), 308 patients experienced either disease progression or death (Figure 1A). The estimated median PFS was 27.7 months (95% CI: 18.7–24.8), with PFS rates at 2 and 4 years of 45.2% (± 2.4 SEM) and 31.8% (± 3.0 SEM), respectively.

**FIGURE 2:**
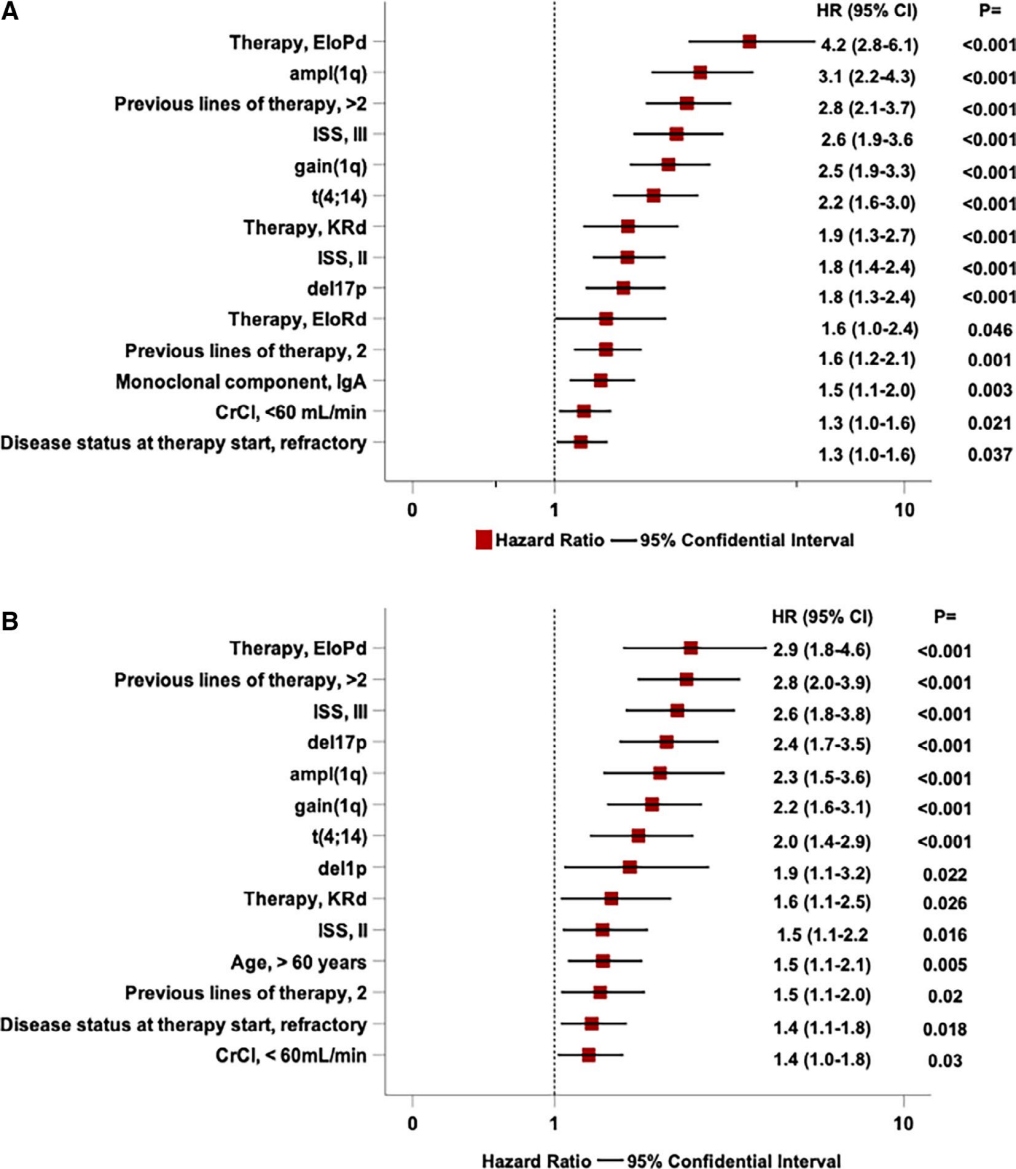
Forest plot of Cox univariable analysis of several clinical, biological, and treatment‐related factors significantly associated with progression‐free survival (A) and overall survival (B).

Figure 2 provides a comprehensive analysis of clinical and genetic factors influencing PFS. The presence of gain(1q) or ampl(1q) was associated with a 2.5‐ and 3.1‐fold increased risk of progression, respectively. Notably, patients lacking +1q alterations exhibited a significantly longer median PFS of 28 months, compared to a median PFS of 8 months for patients with gain(1q) and 7.4 months for those with ampl(1q) (Figure 1B).

Other significant predictors of PFS included the EloPd treatment regimen, which demonstrated a 4.2‐fold increased risk, and the genetic marker *t*(4;14), associated with a 2.2‐fold increased risk. Additionally, extensive prior treatment, that is, previous 2 (HR = 1.6) or > 2 (HR = 2.8) lines of therapy, and advanced disease stages, such as ISS Stage III (HR = 2.6) and II (HR = 1.8), were significantly associated with an increased risk of progression. Age, gender, prior autologous stem cell transplantation, del1p, and *t*(14;16) were not significantly associated with PFS in univariable analyses.

A multivariable Cox regression model, adjusting for all significant variables, showed that gain(1q) (HR = 1.9, 95% CI: 1.4–2.6, *p* < 0.001) and ampl(1q) (HR = 2.2, 95% CI: 1.6–3.2, *p* < 0.001) retained independent prognostic significance. Additional factors that remained independently predictive of increased progression risk included del17p (HR = 1.6, 95% CI: 1.2–2.2, *p* < 0.001), *t*(4;14) (HR = 1.6, 95% CI: 1.1–2.2, *p* = 0.006), CrCl < 60 mL/min (HR = 1.3, 95% CI: 1.0–1.7, *p* = 0.018), ISS Stage II (HR = 1.9, 95% CI: 1.4–2.5, *p* < 0.001), ISS Stage III (HR = 2.7, 95% CI: 2.0–3.7, *p* < 0.001), and treatment with the EloPd regimen (HR = 1.8, 95% CI: 1.1–2.9, *p* = 0.026). In contrast, variables such as the type of monoclonal component, refractory disease status at therapy initiation, and the number of prior therapy lines no longer exhibited significant predictive value for PFS in this model.

### 3.4 Overall Survival

**FIGURE 3:**
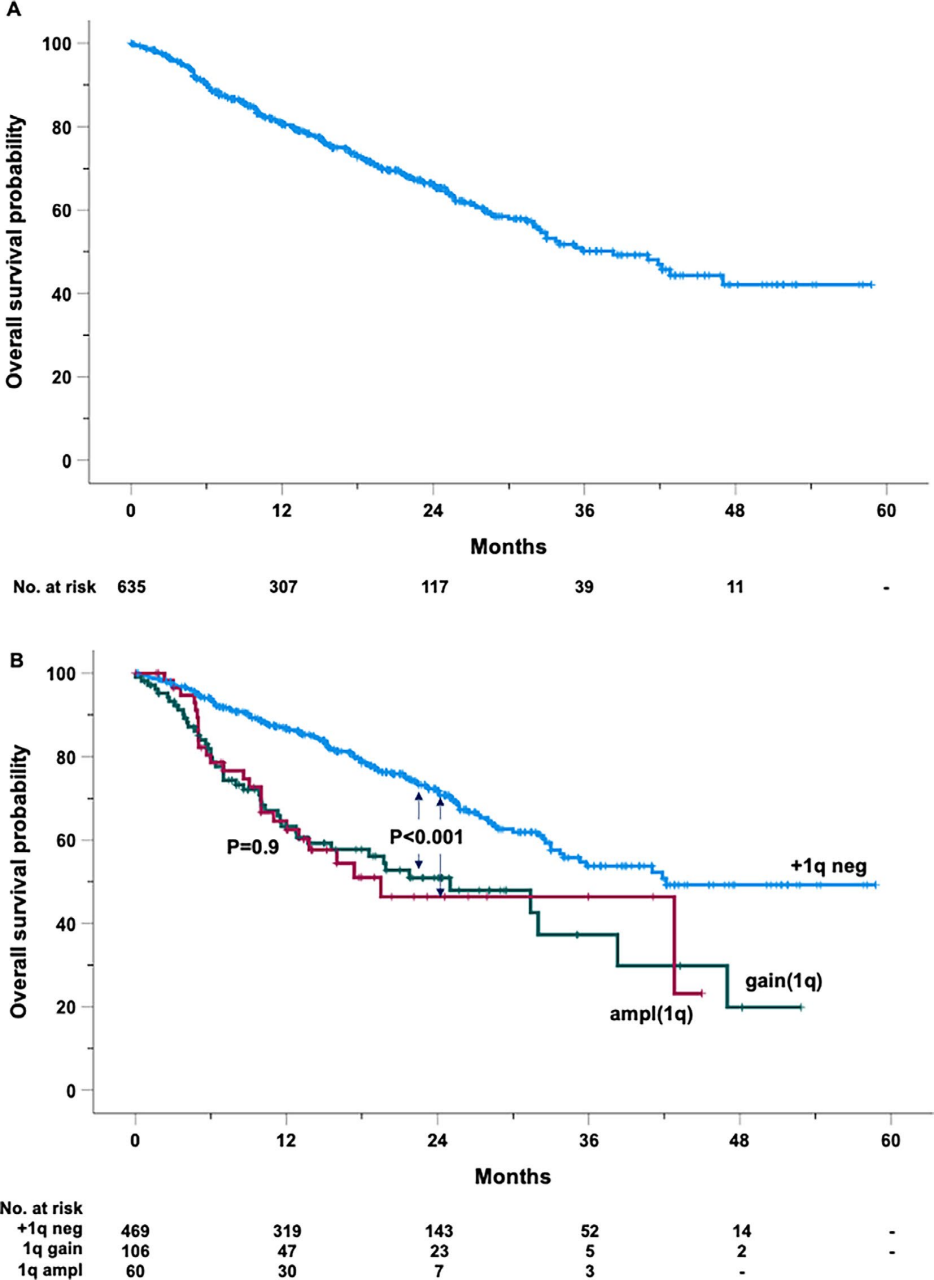
Kaplan–Maier curve of overall survival of the entire cohort (A) and by the absence (+1q neg) or the presence of 1q amplification (ampl1q) or gain gain1q) (B).

During the study period, 200 patients died. The estimated median OS was 38.3 months (95% CI: 32.1–44.4 months). The OS rates at 2 and 4 years were 66.1% and 42.1%, respectively (Figure 3A). In univariable analysis, patients with 1q abnormalities had significantly worse outcomes, with gain(1q) and ampl(1q) associated with a 2.2‐ and 2.3‐fold increase in mortality, respectively (Figure 2B). Median survival differed markedly by genetic subgroup, with those lacking 1q alterations achieving a median OS of 42.2 months, compared to 25 months in patients with gain(1q) and 19.5 months in those with ampl(1q) (Figure 3B). Similarly, the presence of del(17p) resulted in a 2.4‐fold increase in mortality, while del(1p) was linked to a 1.9‐fold increase.

Therapeutic regimens also influenced OS. Among these, patients treated with EloPd experienced a nearly threefold increase in mortality compared to patients treated with DaraRd (reference group). Similarly, those receiving KRd showed a 60% higher risk of mortality compared to the cases receiving DaraRd. The number of prior lines of therapy also critically impacted OS, with patients who had undergone more than two prior lines showing a risk of death nearly tripling, while those with two prior lines experienced a 50% increase in mortality. Additionally, the presence of refractory disease at the initiation of therapy further contributed to poorer survival outcomes (HR = 1.4). Patients with ISS III experienced a 2.6‐fold higher risk of death compared to those with Stage I, while Stage II was associated with a 1.5‐fold increase. Other clinical factors, such as age and renal function, further influenced OS. Older patients, specifically those with a cut‐off value exceeding 60 years of age (Figure S2), showed a 50% higher mortality risk, while impaired renal function, defined as creatinine clearance below 60 mL/min, contributed similarly to poor outcomes. Notably, neither gender nor the monoclonal component type showed a clear association with survival, and the *t*(14;16) translocation lacked prognostic relevance in this cohort.

In the multivariable analysis, gain(1q) and ampl(1q) emerged as independent predictors of increased mortality risk, with HRs of 1.6 (95% CI: 1.1–2.3, *p* = 0.007) and 2.0 (95% CI: 1.3–3.1, *p* = 0.002), respectively. Del(17p) was a strong predictor of poor survival, with a 2.2‐fold increased mortality risk (95% CI: 1.5–3.2, *p* < 0.001), and del(1p) conferred a 1.8‐fold increase in mortality risk (95% CI: 1.02–3.14, *p* = 0.042). The *t*(4;14) translocation was associated with a 1.6‐fold increased mortality risk (95% CI: 1.1–2.4, *p* = 0.022). Moreover, patients with more than two prior lines of therapy (HR 2.1, 95% CI: 1.4–3.3, *p* < 0.001), age ≥ 60 years (HR 1.5, 95% CI: 1.1–2.0, *p* = 0.02), refractory disease at therapy initiation (HR 1.5, 95% CI: 1.1–2.0, *p* = 0.016), and Stage III by ISS (HR 2.5, 95% CI: 1.7–3.7, *p* < 0.001) remained significantly associated with OS in the adjusted model. Conversely, ISS Stage II (*p* = 0.1), two prior lines of therapy (*p* = 0.056), and impaired renal function (CrCl < 60 mL/min) (*p* = 0.08), though suggestive of worse outcomes, did not reach statistical significance. Notably, treatment with either EloPd or KRd compared to DaraRd (reference group) did not demonstrate a significant association with OS in this analysis.

### 3.5 Impact of Concurrent Cytogenetic Abnormalities on Outcomes in +1q Patients

We conducted an in‐depth evaluation of the prognostic significance of +1q abnormalities, both as standalone alterations and in combination with other high‐risk cytogenetic abnormalities, for PFS (Figure S3A,B) and OS (Figure S4A–C). Interactions were specifically analyzed with *t*(4;14) and del17p, which were significant in the multivariable model for PFS, and with *t*(4;14), del17p, and del1p, which were significant in the multivariable model for OS.

Individually, both gain(1q) and ampl(1q) were associated with significantly shorter PFS and OS compared to the reference group (“none”) in the absence of co‐occurring cytogenetic abnormalities. This was demonstrated for *t*(4;14) (Figure S3A for PFS; Figure S4A for OS), del17p (Figure S3B for PFS; Figure S4B for OS), and del1p (Figure S4C for OS).

Although the limited number of cases with combined +1q and other cytogenetic abnormalities limited sample sizes, a clear trend emerged: the presence of +1q in conjunction with high‐risk abnormalities consistently correlated with worse PFS and OS outcomes. However, these associations were not always statistically significant, likely due to limited statistical power stemming from the small number of cases with these combinations.

Patients were further stratified into four groups based on the number of cytogenetic abnormalities, considering those significant in the multivariable analyses for PFS and OS: no‐hit (no abnormalities), single‐hit (one abnormality), double‐hit (two abnormalities), and triple‐hit (three abnormalities). Median PFS varied significantly across these groups, with estimates of 30.5 months (95% CI: 25.4–35.6), 11.0 months (95% CI: 8.3–13.7), 5.0 months (95% CI: 4.5–5.5), and 4.0 months (95% CI: 2.5–7.8), respectively (Figure S3C). PFS was significantly different, observed between the no‐hit and single‐hit groups (*p* < 0.001) and between the single‐hit and double‐hit groups (*p* < 0.001). However, the difference in PFS between the double‐hit and triple‐hit groups was not statistically significant (*p* = 0.7).

Consistent with the PFS findings, OS also differed significantly across these groups. While the median OS was not reached in the no‐hit group, the estimated median OS values were 32 months (95% CI: 21.9–42.1), 13.8 months (95% CI: 3.4–24.2), and 6.4 months (95% CI: 4.6–8.2) for the single‐hit, double‐hit, and triple‐hit groups, respectively (Figure S4D). Notably, statistically significant differences were observed across all cytogenetic groups (*p* < 0.001 for no‐hit versus single‐hit; *p* = 0.026 for single‐hit versus double‐hit; and *p* = 0.004 for double‐hit versus triple‐hit), underscoring the impact of cumulative cytogenetic abnormalities on OS outcomes.

## 4 Discussion

The exploration of +1q abnormalities in MM, particularly in RRMM settings, remains significantly underrepresented in clinical research. A recent comprehensive review of 124 screened clinical trials found that only 29 studies reported information on +1q abnormalities, and of these, only eight provided information on the RRMM setting [33]. Data unavailability becomes even more pronounced in real‐world settings, where cytogenetic testing for +1q abnormalities is uncommon, especially in RRMM.

The findings of this study enhance understanding of the predictive and prognostic role of +1q abnormalities, bridging the knowledge gap in the real‐world RRMM setting. These genetic alterations—encompassing both gain(1q) and ampl(1q)—emerged as critical drivers of adverse prognosis, impacting response quality, PFS, and OS. +1q abnormalities were identified in over a quarter of patients in whom cytogenetic data were available, reflecting their high prevalence in RRMM and their significant contribution to the disease's clinical heterogeneity. From a clinical perspective, the significantly lower rates of deep responses in gain(1q) (9.4%) and ampl(1q) (11.6%) groups compared to patients without these abnormalities (20.2%) highlight the challenge of managing these patients with current salvage treatment. The ordinal logistic regression multivariable analysis reinforces the independent prognostic value of +1q alterations, even when accounting for other critical variables such as ISS stage, *t*(4;14), and del17p. Furthermore, the varying impact of therapeutic regimens on +1q abnormalities warrants further investigation. Logistic regression analysis indicated that patients with gain(1q) or ampl(1q) had significantly lower odds of achieving ≥ CR regardless of the approved therapy used, suggesting that the underlying biology of these high‐risk features limits the efficacy of regimens analyzed in this cohort. After adjustment in the logistic model, DaraRd and KRd were associated with higher response rates compared to EloPd and EloRd, which showed a remarkable 70% and 43% reduction in odds to achieving ≥ CR relative to DaraRd.

Isatuximab is currently approved in combination with Pd in patients who have received at least two prior lines of therapies and in combination with Kd in patients who have received at least one prior line of therapy [38], based on results from the ICARIA‐MM [39, 40] and IKEMA [41, 42] trials, respectively. A recent analysis investigated the outcome of patients harboring +1q abnormalities enrolled in ICARIA‐MM and IKEMA to assess the efficacy of isatuximab in this subset of patients [43]. The study demonstrated the efficacy of the anti‐CD38 monoclonal antibody over the control arm in terms of OS and PFS regardless of the 1q copy number or the presence of other high‐risk cytogenetic abnormalities [43]. Additionally, the Kaplan–Meier curves for PFS and OS in the Isa‐Pd group revealed overlapping outcomes between patients with and without +1q abnormalities, underscoring the comparable efficacy of isatuximab‐based therapies in these cytogenetic subgroups [43]. These findings support the potential of isatuximab‐containing regimens as a viable treatment option for patients harboring +1q abnormalities. Notably, a recent real‐world analysis of isatuximab‐based triplet regimens reported that the presence of +1q abnormalities did not independently predict worse outcomes in a multivariable model [44].

Similarly, there is limited real‐world evidence regarding the use of selinexor (S) [45], alone or in combination with bortezomib and dexamethasone (Vd), despite the encouraging results reported from the Phase III BOSTON trial [46, 47]. The latter demonstrated that the SVd triplet regimen was associated with improved PFS in patients harboring amp(1q), though with borderline significance (HR: 0.63, 95% CI: 0.34–1.17, *p* = 0.07), consistent with findings observed in the overall study population. Recently, Ehsan et al. conducted an observational retrospective single‐center study including RRMM patients with high‐risk cytogenetics, including gain(1q), treated with the selinexor‐based regimen. The analysis suggests that selinexor‐based regimens lead to similar outcomes among RRMM patients with high‐risk and standard‐risk cytogenetics [48].

These findings suggest that both isatuximab‐based regimens and SVd represent viable therapeutic options in real‐world practice, especially for patients harboring +1q abnormalities. However, comprehensive real‐world data assessing the performance of both combinations in patient cohorts harboring +1q abnormalities remain limited. Further studies are necessary to validate the efficacy observed in clinical trials and to confirm the favorable outcomes in broader, unselected real‐world populations.

The significant disparities in survival outcomes between patients with and without +1q abnormalities reinforce the pivotal prognostic role of +1q alterations, still underscoring the adverse biological and clinical implications of these genetic changes. Patients with +1q‐negative disease demonstrated a median PFS of 28 months, more than triple that observed in those with gain(1q) (8 months) or ampl(1q) (7.4 months). Similarly, the median survival for patients with gain(1q) (25 months) and ampl(1q) (19.5 months) was significantly shorter than the 42.2 months observed in +1q‐negative patients. This stark difference in PFS and OS was maintained in the multivariable models, suggesting that +1q alterations not only accelerate disease progression but also diminish the effectiveness of subsequent lines of therapy.

Notably, together with +1q alterations, independent prognostic roles were played by other cytogenetic aberrations such as del17p and *t*(4;14). To explore the role of the presence of multiple genetic alterations in this study, patients were stratified into four groups based on the number of cytogenetic abnormalities, providing a quantitative approach to assessing the prognostic impact of these alterations. By clustering patients into no‐hit, single‐hit, double‐hit, and triple‐hit categories, the analysis demonstrates that increasing cytogenetic complexity correlates with poorer outcomes in both PFS and OS. As expected, in the multivariable model, clustering based on the number of cytogenetic abnormalities preserved both predictive (*p* < 0.001 for PFS) and prognostic (*p* < 0.001 for OS) significance (data not shown). While effective in identifying trends related to increasing genetic burden, this approach may overlook the impact of specific alterations. By focusing on the number of abnormalities rather than their type or combination, it may miss critical insights into how distinct genetic alterations influence prognosis. Nonetheless, clustering based on the number of abnormalities remains a valuable tool for assessing overall risk.

It is noteworthy that the *t*(14;16) translocation did not emerge as a significant factor in any of the outcome evaluations in this study. This finding contrasts with the R‐ISS [21], where *t*(14;16) is included as a high‐risk cytogenetic abnormality but aligns with the R2‐ISS [25], which excludes *t*(14;16) as an independent prognostic marker. A possible explanation for this observation is the incorporation of +1q alterations into the analysis, a variable that likely supersedes the prognostic role of *t*(14;16), as observed in the R2‐ISS [25]. These results suggest that, similar to NDMM, the *t*(14;16) translocation does not add prognostic value in RRMM. This highlights the limited clinical utility of *t*(14;16) as a standalone marker in the context of modern risk stratification models, both in NDMM and RRMM settings. The findings further emphasize the importance of integrating +1q alterations and other robust markers to refine prognostic tools and improve patient stratification and outcomes. Indeed, previous studies [44, 47] have already demonstrated the utility of the therapeutic approach with more efficient novel agents, such as isatuximab and selinexor, in this high‐risk subset of patients, potentially effective as bridging therapy for even more effective therapeutic platforms including bispecific antibodies and CAR‐T therapies.

Furthermore, in this study, ISS continued to demonstrate its strong prognostic value in predicting response, PFS, and OS.

The limited availability of FISH analysis for +1q abnormalities in 42.1% of the cohort (635/1556 cases) constitutes a potential limitation of this study. Nevertheless, in order to mitigate the impact of missing cases, we found that the baseline characteristics of patients with and without available +1q data were comparable, observing no significant differences across key variables. This finding suggests that the subgroup with available +1q abnormality analysis is representative of the overall cohort, hence minimizing the constraints of the retrospective analyses. However, the relatively low incidence of +1q alterations observed in this study, in part justified by a high positivity cut‐off, could reflect a selection bias due to cases without available analysis, potentially underestimating the true prevalence of these abnormalities.

The lack of detailed data on class‐refractory status (i.e., refractoriness to CD38 monoclonal antibodies, proteasome inhibitors, or immunomodulatory drugs) represents a limitation of our study. While refractoriness to the last line of therapy was considered, the absence of a more refined classification of treatment resistance may have impacted the observed outcomes, especially when comparing different triplet regimens. This limitation is inherent to real‐world data, where treatment decisions are influenced by prior drug exposures and evolving therapeutic landscapes. For instance, patients receiving Elo‐Pd may have been more likely to be lenalidomide‐refractory compared to those treated with Dara‐Rd, potentially affecting response rates and survival outcomes. Future studies should incorporate class‐refractory status into prognostic models to improve treatment stratification in real‐world settings.

In conclusion, the role of +1q abnormalities in MM, particularly in the RRMM setting, remains insufficiently explored in clinical research, further compounded by the limited use of cytogenetic testing in real‐world practice. In this scenario, our data provide an initial contribution toward addressing this knowledge gap, underscoring the critical role of +1q alterations as both predictive and prognostic markers in RRMM. Additionally, our findings highlight the critical importance of +1q abnormalities in RRMM even in the real‐world setting, providing valuable insights into risk stratification and treatment decision‐making. The study also reinforces the need for comprehensive cytogenetic profiling and a more refined approach to prognostic evaluation, integrating both genetic and clinical factors to optimize patient outcomes in this complex disease landscape. In the future, it will be essential to implement these advanced genotyping technologies, such as NGS, not only in research [49] but also in real‐world clinical settings. However, in clinical practice, molecular information must not only demonstrate prognostic value but also guide specific therapeutic decisions, ensuring its practical utility in tailoring treatment strategies for MM both at diagnosis and at relapse.

## Author Contributions

F.M., E.A.M., S.M., M.G., F.D.R., E.B., A.N., F.M., and P.M. designed the study. M.G., G.T., G.D., and F.M. performed statistical analysis. M.G., M.O., R.Z., S.B., N.G., C.C., M.B., A.G., N.S., A.M.Q., G.B., A.B., C.C., G.M.C., R.F., V.B., G.T., D.D., F.P., A.G., A.S., E.A., A.R., F.L., C.D.M., M.T.P., N.B., C.C., E.Z., S.P., M.M., A.M., R.D.P., E.V., A.B., F.F., R.M., L.P., I.D.V., G.F., C.C., K.M., A.P.F., G.M., A.S., G.R., E.A., M.Q., C.S.C., S.P., M.M., F.F., A.C., M.P., V.M., S.M., L.P., and V.V.F. analyzed and interpreted data. E.A.M., M.G., S.M., and F.M. wrote the manuscript. All authors gave final approval.

## Ethics Statement

The study protocol was reviewed and approved by the Institutional Ethics Committees in accordance with the principles of the Declaration of Helsinki.

## Consent

All patients enrolled have signed the informed consent.

## Conflicts of Interest

The authors declare no conflicts of interest.

## Supporting information


**Figure S1.** Flowchart indicating the selection process of cases meeting the inclusion criteria for the study.

**Figure S2.** Receiver operating characteristic (ROC) analysis of age to identify patients who died. The dashed line represents the reference line of prognostic usefulness.

**Figure S3.** Kaplan–Meier curves of progression‐free survival (PFS) stratified by cytogenetic alterations and risk Groups. (A) PFS stratified by the combination of +1q alterations and *t*(4;14). (B) PFS stratified by the combination of 1q alterations and del(17p). (C) PFS categorized by cytogenetic risk groups: no hit, single hit, double hit, and triple hit.

**Figure S4.** Kaplan–Meier curves of overall survival (OS) stratified by cytogenetic alterations and risk groups. (A) OS stratified by the combination of +1q alterations and *t*(4;14). (B) OS stratified by the combination of +1q alterations and del(17p). (C) OS stratified by the combination of +1q alterations and del1q. Panel D. OS categorized by cytogenetic risk groups: no hit, single hit, double hit, and triple hit.

**Data S1.** Supporting Information.

## Data Availability

The data that support the findings of this study are available from the corresponding author upon reasonable request.
