# Supplementary figures and images for: Prognostic Significance of +1q Alterations in Relapsed/Refractory Multiple Myeloma Treated With Daratumumab‐, Elotuzumab‐, and Carfilzomib‐Based Triplet Regimens: A Multicenter Real‐World Analysis of 635 Patients
Source: Eur J Haematol. 2025 Mar 19;115(1):16–28. doi: 10.1111/ejh.14413 (PMC12134713; doi:10.1111/ejh.14413)

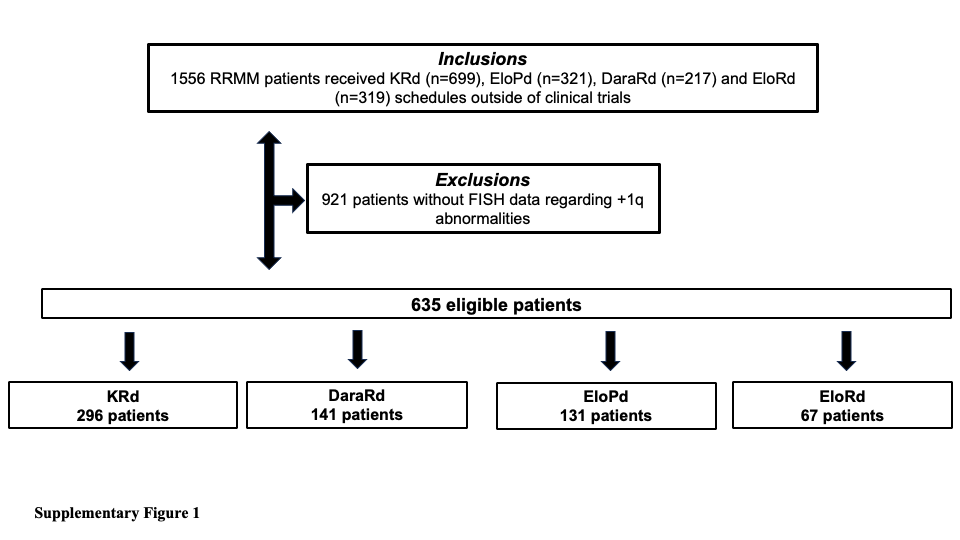

Supplement: Supplementary file 1 — Figure S1. Flowchart indicating the selection process of cases meeting the inclusion criteria for the study. [file EJH-115-16-s001.tiff]

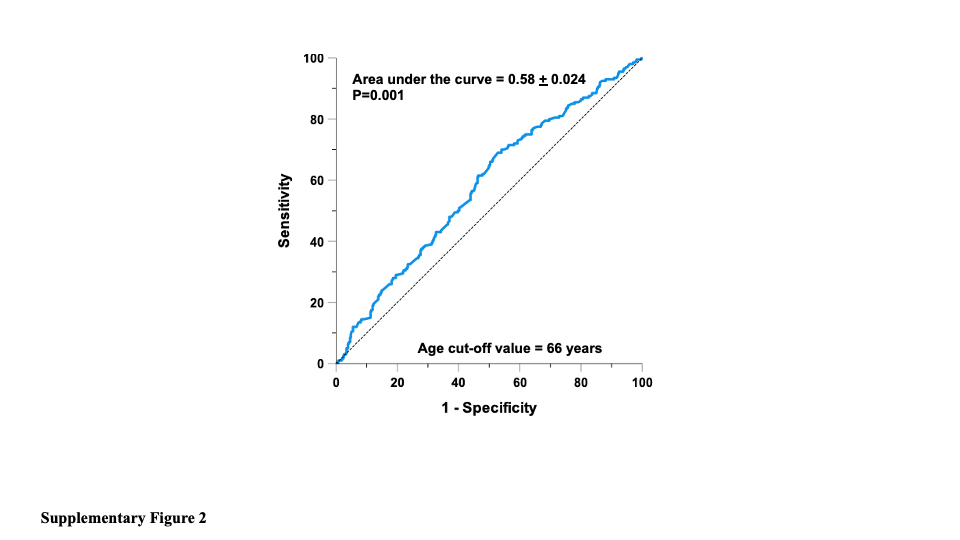

Supplement: Supplementary file 2 — Figure S2. Receiver operating characteristic (ROC) analysis of age to identify patients who died. The dashed line represents the reference line of prognostic usefulness. [file EJH-115-16-s003.tiff]

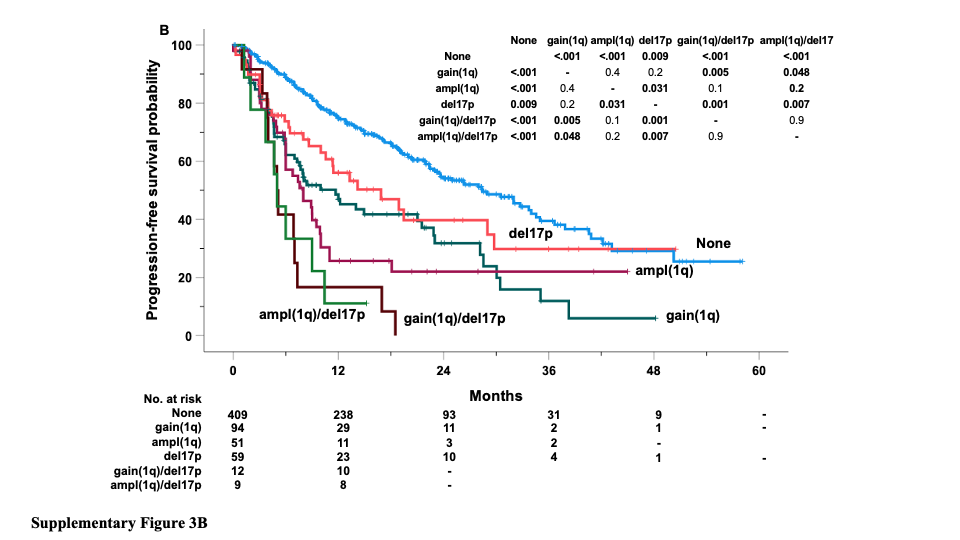

Supplement: Supplementary file 3 — Figure S3. Kaplan–Meier curves of progression‐free survival (PFS) stratified by cytogenetic alterations and risk Groups. (A) PFS stratified by the combination of +1q alterations and t(4;14). (B) PFS stratified by the combination of 1q alterations and del(17p). (C) PFS categorized by cytogenetic risk groups: no hit, single hit, double hit, and triple hit. [file EJH-115-16-s005.zip › ejh14413-sup-0004-FigureS3B.tiff]

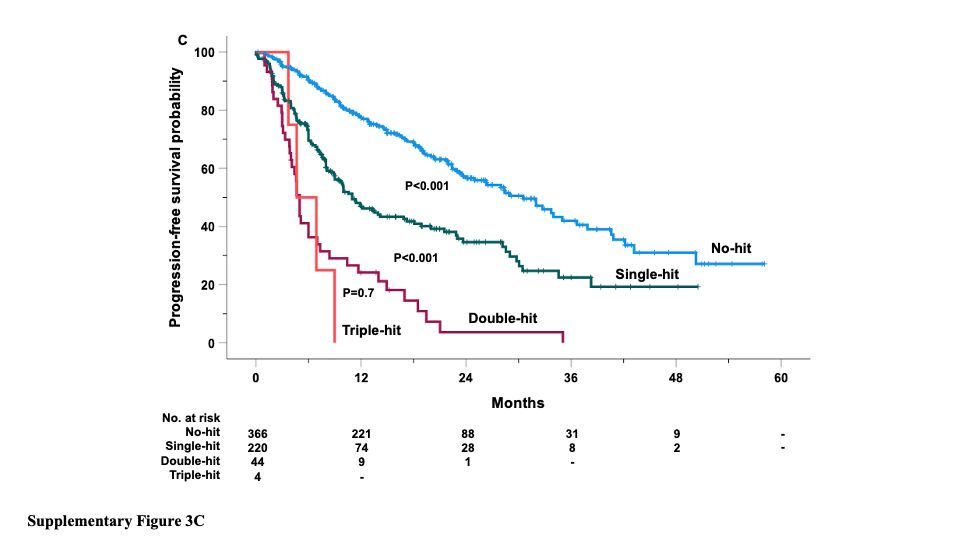

Supplement: Supplementary file 3 — Figure S3. Kaplan–Meier curves of progression‐free survival (PFS) stratified by cytogenetic alterations and risk Groups. (A) PFS stratified by the combination of +1q alterations and t(4;14). (B) PFS stratified by the combination of 1q alterations and del(17p). (C) PFS categorized by cytogenetic risk groups: no hit, single hit, double hit, and triple hit. [file EJH-115-16-s005.zip › ejh14413-sup-0005-FigureS3C.tiff]

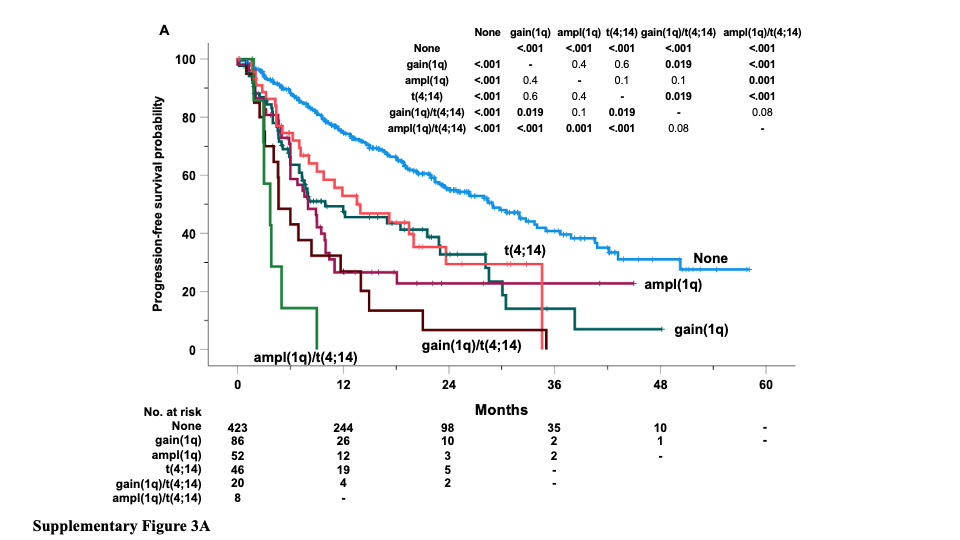

Supplement: Supplementary file 3 — Figure S3. Kaplan–Meier curves of progression‐free survival (PFS) stratified by cytogenetic alterations and risk Groups. (A) PFS stratified by the combination of +1q alterations and t(4;14). (B) PFS stratified by the combination of 1q alterations and del(17p). (C) PFS categorized by cytogenetic risk groups: no hit, single hit, double hit, and triple hit. [file EJH-115-16-s005.zip › ejh14413-sup-0003-FigureS3A.tiff]

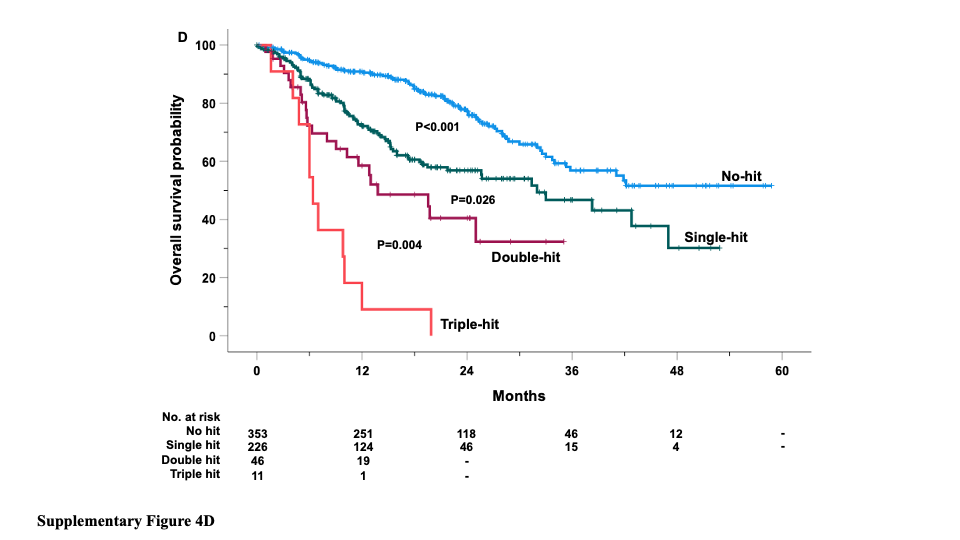

Supplement: Supplementary file 4 — Figure S4. Kaplan–Meier curves of overall survival (OS) stratified by cytogenetic alterations and risk groups. (A) OS stratified by the combination of +1q alterations and t(4;14). (B) OS stratified by the combination of +1q alterations and del(17p). (C) OS stratified by the combination of +1q alterations and del1q. Panel D. OS categorized by cytogenetic risk groups: no hit, single hit, double hit, and triple hit. [file EJH-115-16-s002.zip › ejh14413-sup-0009-FigureS4D.tiff]

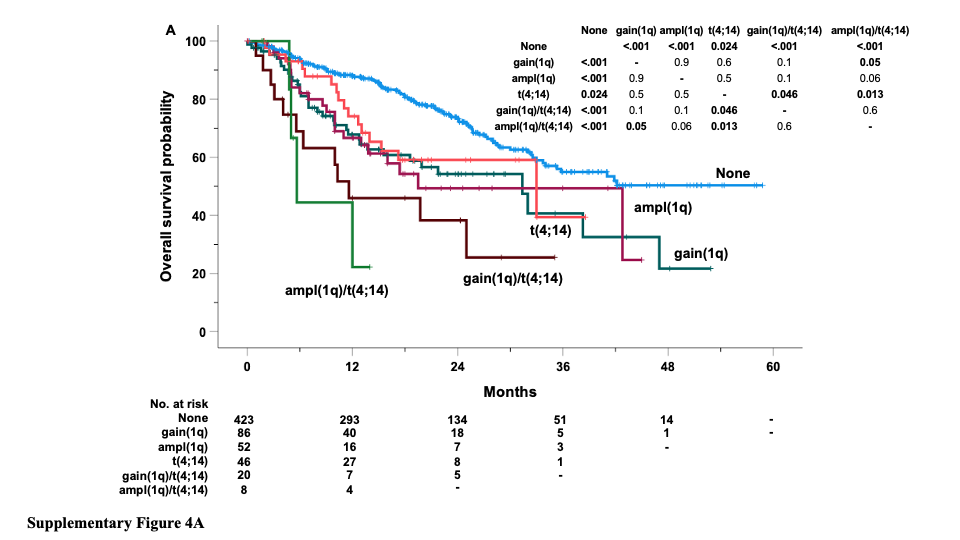

Supplement: Supplementary file 4 — Figure S4. Kaplan–Meier curves of overall survival (OS) stratified by cytogenetic alterations and risk groups. (A) OS stratified by the combination of +1q alterations and t(4;14). (B) OS stratified by the combination of +1q alterations and del(17p). (C) OS stratified by the combination of +1q alterations and del1q. Panel D. OS categorized by cytogenetic risk groups: no hit, single hit, double hit, and triple hit. [file EJH-115-16-s002.zip › ejh14413-sup-0006-FigureS4A.tiff]

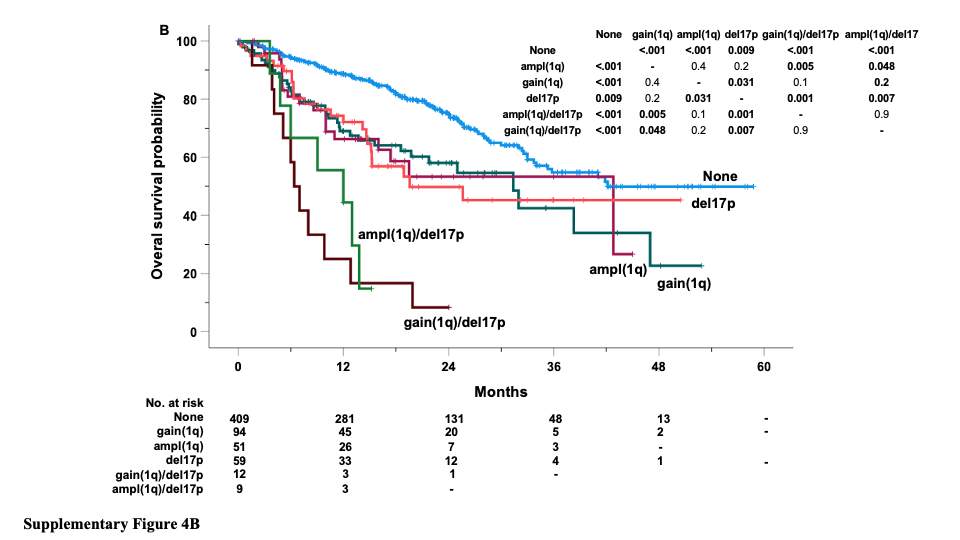

Supplement: Supplementary file 4 — Figure S4. Kaplan–Meier curves of overall survival (OS) stratified by cytogenetic alterations and risk groups. (A) OS stratified by the combination of +1q alterations and t(4;14). (B) OS stratified by the combination of +1q alterations and del(17p). (C) OS stratified by the combination of +1q alterations and del1q. Panel D. OS categorized by cytogenetic risk groups: no hit, single hit, double hit, and triple hit. [file EJH-115-16-s002.zip › ejh14413-sup-0007-FigureS4B.tiff]

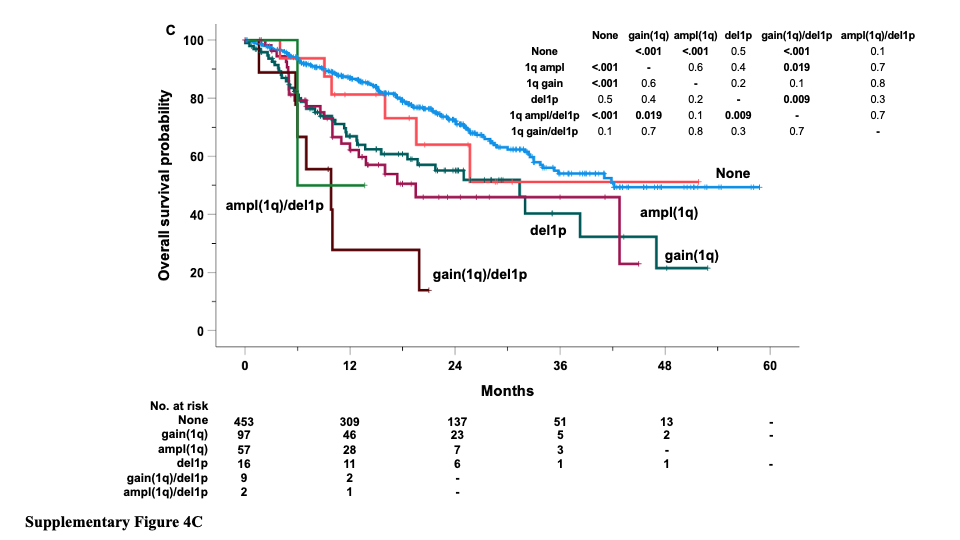

Supplement: Supplementary file 4 — Figure S4. Kaplan–Meier curves of overall survival (OS) stratified by cytogenetic alterations and risk groups. (A) OS stratified by the combination of +1q alterations and t(4;14). (B) OS stratified by the combination of +1q alterations and del(17p). (C) OS stratified by the combination of +1q alterations and del1q. Panel D. OS categorized by cytogenetic risk groups: no hit, single hit, double hit, and triple hit. [file EJH-115-16-s002.zip › ejh14413-sup-0008-FigureS4C.tiff]
