# Supplementary material for: Prognostic Significance of +1q Alterations in Relapsed/Refractory Multiple Myeloma Treated With Daratumumab‐, Elotuzumab‐, and Carfilzomib‐Based Triplet Regimens: A Multicenter Real‐World Analysis of 635 Patients
Source: Eur J Haematol. 2025 Mar 19;115(1):16–28. doi: 10.1111/ejh.14413 (PMC12134713; doi:10.1111/ejh.14413)
Supplement: Supplementary file 5 — Data S1. Supporting Information. [file EJH-115-16-s004.docx]

**SUPPLEMENTARY APPENDIX CONTENTS**

**Prognostic Significance of +1q Alterations in Relapsed/Refractory Multiple Myeloma Treated with Daratumumab-, Elotuzumab-, and Carfilzomib-Based Triplet Regimens: A Multicenter Real-World Analysis of 635 Patients**

**Fortunato Morabito^1*^, Enrica Antonia Martino^2*^, Monica Galli^3^, Massimo Offidani^4^, Renato Zambello^5^, Sara Bringhen^6^, Nicola Giuliani^7^, Catello Califano^8^, Marino Brunori^9^, Alfredo Gagliardi^10^, Nicola Sgherza^11^, Angela Maria Quinto^12^, Gregorio Barilà^13^, Angelo Belotti^14^, Claudio Cerchione^15^, Gloria Margiotta Casaluci^16^, Raffaele Fontana^17^, Velia Bongarzoni^18^, Giuseppe Tarantini^19^, Daniele Derudas^20^, Francesca Patriarca^21^, Alessandro Gozzetti^22^, Adelina Sementa^23^, Elisabetta Antonioli^24^, Angela Rago^25^, Flavia Lotti^26^, Claudio De Magistris^27^, Maria Teresa Petrucci^28^, Loredana Pettine^27^, Niccolò Bolli^27^, Concetta Conticello^29^, Elena Zamagni^30,31^, Salvatore Palmieri^32^, Maurizio Musso^33^, Anna Mele^34^, Roberta Della Pepa^35^, Ernesto Vigna^2^, Antonella Bruzzese^2^, Francesca Fazio^28^, Roberto Mina^6^, Laura Paris^3^, Iolanda Donatella Vincelli^36^, Giuliana Farina^37^, Clotilde Cangialosi^38^, Katia Mancuso^30,31^, Antonietta Pia Falcone^39^, Giuseppe Mele^40^, Antonello Sica^41^, Sonia Morè^4^, Giovanni Reddiconto^42^, Giovanni Tripepi^43^, Graziella D’Arrigo^43^, Emiliano Barbieri^44,45^, Micol Quaresima^44^, Claudio Salvatore Cartia^46^, Sara Pezzatti^47^, Magda Marcatti^48^, Francesca Farina^48^, Anna Cafro^49^, Michele Palumbo^50^, Valeria Masoni^50^, Virginia Valeria Ferretti^51^, Francesco Di Raimondo^30^, Pellegrino Musto^11^, Antonino Neri^52^, Silvia Mangiacavalli^46^ˆ, Massimo Gentile^2,53^ˆ**

***Equally contributed as first authors**

**^Equally contributed as senior authors**

^1^Gruppo Amici Dell'Ematologia Foundation-GrADE, Reggio Emilia ^2^Hematology Unit AO of Cosenza, Cosenza, Italy; ^3^Hematology and Bone Marrow Transplant Unit, ASST Papa Giovanni XXIII, Bergamo; ^4^Hematology Unit, AOU delle Marche, Ancona; ^5^Department of Medicine (DIMED), Hematology and Clinical Immunology, Padua University School of Medicine, Padova; ^6^Division of Hematology, AOU Città della Salute e della Scienza di Torino, University of Torino, Torino; ^7^Hematology Unit, Parma University Hospital, Parma; ^8^Onco-Hematology Unit, "A. Tortora" Hospital, Pagani; ^9^Internal Medicine, Ospedale Santa Croce, Fano; ^10^Hematology, "Santa Maria di Loreto Nuovo" Hospital, Naples; ^11^Unit of Hematology and Stem Cell Transplantation, AOUC Policlinico, Bari; ^12^Haematology and Transplant Unit, IRCCS - Istituto Tumori "Giovanni Paolo II", Bari; ^13^Hematology Unit, Ospedale San Bortolo, Vicenza; ^14^Hematology Unit, A.O. Spedali Civili, Brescia; ^15^Department of Hematology, IRCCS Istituto Scientifico Romagnolo per lo Studio e la Cura dei Tumori (IRST), Meldola, Forlì-Cesena; ^16^Division of Hematology, Department of Translational Medicine, University of Eastern Piedmont, Novara; ^17^Hematology and Transplant Center, University Hospital "San Giovanni di Dio e Ruggi d'Aragona", Salerno; ^18^UOC of Hematology San Giovanni-Addolorata Hospital Rome; ^19^Hematology Unit, "Dimiccoli" Hospital, Barletta (BAT); ^20^Department of Hematology, Businco Hospital, Cagliari; ^21^Hematology, DMED, University of Udine, University Hospital of Friuli Centrale, Udine; ^22^Department of Medicine, Surgery and Neurosciences, University of Siena Policlinico S Maria alle Scotte, Siena; ^23^UOC Ematologia, Ospedale San Giuseppe Moscati, Avellino; ^24^Haematology Unit Careggi University Hospital, Florence; ^25^UOSD Ematologia ASL Roma 1, Rome; ^26^Institute of Hematology, TMO Azienda Universitaria-Ospedaliera Santa Maria della Misericordia di Perugia, Perugia; ^27^Hematology Unit, Fondazione IRCCS Ca' Granda Ospedale Maggiore Policlinico, Milan; ^28^Department of Translational and Precision Medicine, Hematology Azienda Policlinico Umberto I Sapienza University of Rome; ^29^Division of Hematology, Azienda Policlinico-OVE, University of Catania, Catania; ^30^IRCCS Azienda Ospedaliero-Universitaria di Bologna, Istituto di Ematologia "Seràgnoli", Bologna; ^31^Dipartimento di Medicina Specialistica, Diagnostica e Sperimentale, Università di Bologna, Bologna; ^32^Hematology Unit, Ospedale Cardarelli, Napoli; ^33^UOC OncoEmatologia e TMO, Dipartimento Oncologico, La Maddalena, Palermo; ^34^Department of Hematology and Bone Marrow Transplant, Hospital Card. G. Panico, Tricase, Lecce; ^35^Hematology, AUOP "Federico II", Naples; ^36^Hematology Unit, Department of Hemato-Oncology and Radiotherapy, Great Metropolitan Hospital "Bianchi-Melacrino-Morelli", Reggio Calabria; ^37^UOC Ematologia a Indirizzo Oncologico, AORN “Sant’Anna e San Sebastiano”, Caserta; ^38^UOC Ematologia A. O. Ospedali Riuniti Villa Sofia-Cervello, Palermo; ^39^Department of Hematology and Bone Marrow Transplant, IRCCS Casa Sollievo della Sofferenza, San Giovanni Rotondo; ^40^Department of Hematology, Hospital Perrino, Brindisi, Italy; ^41^Onco-Hematology, AOU "Vanvitelli", Naples, Italy. ^42^Department of Hematology, Hospital Vito Fazzi, Lecce; ^43^CNR-IBIM, Clinical Epidemiology and Physiopathology of Renal Diseases and Hypertension of Reggio Calabria, Reggio Calabria; ^44^Hematology Unit, Azienda USL-IRCCS di Reggio Emilia, Emilia Romagna; ^45^Clinical and Experimental Medicine PhD Program, University of Modena and Reggio Emilia, Modena, Italy; ^46^Division of Hematology, IRCCS Fondazione Policlinico San Matteo, Pavia; ^47^Division of Hematology, San Gerardo Hospital, Monza; ^48^Division of Hematology and Bone Marrow Transplant Unit, IRCCS San Raffaele Scientific Institute, Milan; ^49^Division of Hematology and Bone Marrow Transplant Unit, IRCCS San Raffaele Scientific Institute, Milan; ^50^Department of Molecular Medicine, University of Pavia, Pavia; ^51^Clinical Epidemiology and Biostatistics Service, Fondazione IRCCS Policlinico San Matteo, Pavia; ^52^Scientific Directorate IRCCS of Reggio Emilia, I 42123 Reggio Emilia, Emilia Romagna; ^53^Department of Pharmacy, Health and Nutritional Science, University of Calabria, Rende.

**Correspondence: Massimo Gentile, MD, Hematology Unit, AO of Cosenza, Italy; 87100 Cosenza, Italy; e-mail: massimo.gentile@unical.it; ph: +39-0984-681329; fax: +39-0984-681329; Enrica Antonia Martino, Hematology Unit, AO of Cosenza, Italy; 87100 Cosenza, Italy; e-mail: enricaantoniamartino@gmail.com; ph: +39-0984-681329; fax: +39-0984-681329;**

**Competing interests**: Nothing to disclose

**Supplementary Tables**

**Supplementary Figure Legends**

**Supplementary Table 1. Main characteristics of patients at salvage therapy initiation according to 1q analysis availability.**

|  | **No. of cases without 1q analysis available (%)** | **No. of cases with 1q analysis available (%)** | **P value** |
| --- | --- | --- | --- |
| **Age, (years)**  <70  ≥70 | 518 (55.7)  403 (44.3) | 367 (57.8)  268 (42.2) | NS |
| **Sex**  Male  Female | 478 (51.9)  443 (48.1) | 334 (52.6)  301 (47.4) | NS |
| **Paraproteins (isotype)**  Immunoglobulin G  Immunoglobulin A  Immunoglobulin D  Immunoglobulin M  Light chain only  No secretory | 526 (57.1)  213 (23.1)  18 (1.9)  7 (0.8)  167 (18.1)  10 (1.1) | 375 (59.1)  136 (21.4)  9 (1.4)  4 (0.6)  104 (16.4)  7 (1.1) | NS |
| **CrCL (mL/min)**  ≥60  <60 | 588 (63.8)  333 (36.2) | 394 (62)  241 (38) | NS |
| **Stage ISS, (%)**  I  II  III | 347 (37.7)  354 (38.4)  220 (23.9) | 250 (39.4)  249 (39.2)  136 (21.4) | NS |
| **Previous lines of therapy**  1  2  >2 | 524 (56.9)  217 (23.6)  180 (19.5) | 377 (59.4)  148 (23.3)  110 (17.3) | NS |
| **Previous ASCT**  No  Yes | 513 (55.7)  408 (44.3) | 366 (57.6)  269 (42.4) | NS |
| **Disease status**  Relapse  Refractory  Missing | 466 (50.5)  348 (37.8)  107 (11.6) | 310 (48.8)  234 (36.8)  91 (14.3) | NS |

**Abbreviations**: NS = not significant

| **Supplementary Table 2.** Main clinical-hematological and biological characteristics resulted significantly associated with response. | | | | | | |
| --- | --- | --- | --- | --- | --- | --- |
|  |  | **>CR** | **VGPR** | **PR** | **Others** | **P value** |
| **Monoclonal Component** | IgG | 57 (15.2) | 116 (30.9) | 125 (33.3) | 77 (20.5) | 0.037 |
|  | IgA | 21 (15.4) | 47 (34.5) | 30 (22) | 38 (27.9) |  |
|  | Light chain only | 28 (26.9) | 29 (27.9) | 26 (25) | 21 (20.2) |  |
|  | Others | 6 (30) | 5 (25) | 6 (30) | 3 (15) |  |
| **ISS** | I | 52 (20.8) | 81 (32.4) | 71 (28.4) | 46 (18.4) | 0.028 |
|  | II | 40 (16) | 77 (30.9) | 83 (33.3) | 49 (19.6) |  |
|  | III | 20 (14.7) | 39 (28.6) | 33 (24.2) | 44 (32.3) |  |
| **Previous lines of therapies** | 1 line | 86 (22.8) | 140 (37.1) | 102 (27) | 49 (12.9) | <0.001 |
|  | 2 lines | 18 (12.1) | 39 (26.3) | 46 (31) | 45 (30.4) |  |
|  | > 2 lines | 8 (7.2) | 18 (16.3) | 39 (35.4) | 45 (40.9) |  |
| **Last therapy** | DaraRd | 35 (24.8) | 53 (37.5) | 38 (26.9) | 15 (10.6) | <0.001 |
|  | EloRd | 6 (8.9) | 26 (38.8) | 23 (34.3) | 12 (17.9) |  |
|  | KRd | 69 (23.3) | 95 (32) | 78 (26.3) | 54 (18.2) |  |
|  | EloPd | 2 (1.5) | 23 (17.5) | 48 (36.6) | 58 (44.2) |  |
| **t(4‎:14)** | negative | 105 (18.7) | 178 (31.7) | 163 (29) | 115 (20.4) | 0.037 |
|  | positive | 7 (9.4) | 19 (25.6) | 24 (32.4) | 24 (32.4) |  |
| **del17p** | negative | 100 (18) | 180 (32.4) | 162 (29.2) | 112 (20.2) | 0.020 |
|  | positive | 12 (15) | 16 (20) | 25 (31.2) | 27 (33.7) |  |
| **1q alterations** | +1q negative | 95 (20.2) | 161 (34.3) | 132 (28.1) | 81 (17.2) | <0.001 |
|  | 1q gain | 10 (9.4) | 23 (21.6) | 36 (33.9) | 37 (34.9) |  |
|  | 1q ampl | 7 (11.6) | 13 (21.6) | 19 (31.6) | 21 (35.2) |  |

**Supplementary Table** 3 Multivariable ordinal logistic regression model of response

|  | **Odds ratio (95% CI), P value** |
| --- | --- |
| **Monoclonal Component**  IgG  IgA  Other | 1*  1.11 (0.77-1.61), P=0.569  1.57 (1.07-2.31), P=0.021 |
| **°ISS** | 0.78 (0.64-0.95), P=0.013 |
| **°Previous lines of therapy** | 0.64 (0.51-0.81), P<0.001 |
| **Last therapy**  DaraRd  EloPd  EloRd  KRd | 1*  0.30 (0.17-0.51), P<0.001  0.53 (0.31-0.90), P=0.020  0.93 (0.63-1.39), P=0.732 |
| **t(4‎:14)**  negative  positive | 1*  0.72 (0.45-1.14), P=0.16 |
| **del17p**  negative  positive | 1*  0.59 (0.37-0.92), P=0.019 |
| **+1q alterations**  +1q negative  1q gain  1q ampl | 1*  0.49 (0.33-0.73), P<0.001  0.58 (0.35-0.97), P=0.037 |

*Reference category; °1 unit of increase

**Supplementary Figure legend**

**Supplementary Figure 1.** Flowchart indicating the selection process of cases meeting the inclusion criteria for the study.

**Supplementary Figure 2.** Receiver Operating Characteristic (ROC) analysis of age to identify patients who died. The dashed line represents the reference line of prognostic usefulness.

**Supplementary Figure 3.** Kaplan-Meier Curves of Progression-Free Survival (PFS) Stratified by Cytogenetic Alterations and Risk Groups. Panel A. PFS stratified by the combination of +1q alterations and t(4;14). Panel B. PFS stratified by the combination of 1q alterations and del(17p). Panel C. PFS categorized by cytogenetic risk groups: no hit, single hit, double hit, and triple hit.

**Supplementary Figure 4.** Kaplan-Meier Curves of Overall Survival (OS) Stratified by Cytogenetic Alterations and Risk Groups. Panel A. OS stratified by the combination of +1q alterations and *t*(4;14). Panel B. OS stratified by the combination of +1q alterations and del(17p). Panel C. OS stratified by the combination of +1q alterations and del1q. Panel D. OS categorized by cytogenetic risk groups: no hit, single hit, double hit, and triple hit.
